# Longitudinal association between density of retail food stores and body mass index in Mexican school children and adolescents

**DOI:** 10.1038/s41366-023-01273-w

**Published:** 2023-02-15

**Authors:** Yenisei Ramírez-Toscano, Carolina Pérez-Ferrer, Usama Bilal, Amy H. Auchincloss, Tonatiuh Barrientos-Gutierrez

**Affiliations:** 1 Center for Population Health Research, National Institute of Public Health, Cuernavaca, Morelos Mexico; 2 Center for Nutrition and Health Research, National Institute of Public Health, Cuernavaca, Morelos Mexico; 3 National Council for Science and Technology (CONACYT), Ciudad de Mexico, Mexico; 4 Urban Health Collaborative, Drexel Dornsife School of Public Health, Philadelphia, PA USA; 5 Department of Epidemiology and Biostatistics, Drexel Dornsife School of Public Health, Philadelphia, PA USA

**Keywords:** Obesity, Risk factors

## Abstract

**Background:**

Obesity is rapidly increasing in Mexican children and adolescents, while food environments are rapidly changing. We evaluated the association between changes in retail food stores and change in body mass index (BMI) in Mexican children and adolescents.

**Methods:**

Data on 7507 participants aged 5–19 years old came from the Mexican Family Life Survey 2002–2012. Density of food stores at the municipal-level (number of food stores/area in km^2^) came from the Economic Censuses of 1999, 2004 and 2009. We categorized food stores as small food retail (small neighborhood stores, *tiendas de abarrotes* in Mexico), specialty foods, fruit/vegetables, convenience foods, and supermarkets. Associations between change in food stores and change in BMI were estimated using five longitudinal linear fixed-effects regression models (one per type of food store) adjusted for age, parental education, municipal-level socioeconomic deprivation and population density. Density of each food store type was operationalized as quartiles. Analyses were stratified by urbanization.

**Results:**

There was an inverse dose-response association between increases in fruit/vegetable store density and BMI (β = −0.455 kg/m^2^, β = −0.733 kg/m^2^, and β = −0.838 kg/m^2^ in the second, third, and fourth quartile). In non-urban areas, children living in municipalities with the highest density of small food retail stores experienced a reduction in BMI (β = −0.840 kg/m^2^). In urban areas, there was an inverse association between specialty food stores with BMI (β = −0.789 kg/m^2^ in third quartile, and β = −1.204 kg/m^2^ in fourth quartile). We observed dynamic associations with age; results suggested stronger associations in adolescents.

**Conclusions:**

The availability of fruit/vegetable stores may influence a reduction in children and adolescents BMI. These results indicate that policy approaches could be tailored by type of food store – with some consideration for level of urbanization and children’s age.

## Introduction

Overweight and obesity are rapidly increasing in Mexican children and adolescents. During the past 20 years, childhood overweight and obesity has increased nearly one percentage point every two years. In 2018, overweight and obesity prevalence in children 5 to 11 was 36%, while in adolescents it was 38% [1]. Although the association between consumption of high energy-dense foods [2], and sugar-sweetened beverages with obesity has been studied [3], less emphasis has been given to the contextual factors that determine these behaviors. The changes in the prevalence of obesity suggest that large-scale environmental forces and structural drivers are influencing children’s obesity-related behaviors, including diet and physical activity [4].

The food environment could be a key driver of dietary change, as it facilitates the consumption of healthy or unhealthy foods at the population level [5]. By food environment, we refer to the type and availability of food stores, including supermarkets, grocery stores, convenience stores, fruit and vegetable markets or fast food and take-out restaurants, within a community [6]. Previous systematic reviews [7, 8] and reviews of systematic reviews [9, 10] from high-income countries have focused on the association between the food environment, dietary intake and obesity and noted that the evidence has been heterogenous and inconsistent [8–10]. These reviews have recommended the use of study designs that support causal inference (e.g., longitudinal designs), use of reliable measures for food environment, replication in non-US contexts [9], as well as studies focused on children and adolescents [8].

Several studies have analyzed the impact of the food environment on children’s body weight. However, according to a systematic review [7], studies in children have been predominantly cross-sectional, have been conducted in high-income countries, and focused on only one or two types of food establishments. In Mexico, cross-sectional studies reported that a higher number of mobile food vendors outside schools [11] and higher density of supermarkets [12] are associated with higher children’s body mass index (BMI) or a higher prevalence of overweight/obesity; further a study from adult population reported that convenience stores were associated with higher mean BMI [13]. The few studies that have longitudinally examined associations between change in food establishments and change in children’s weight, have been conducted in the US. In the US, less healthy food environments (such as those characterized by a higher density of convenience stores [14] or limited-service restaurants [15–17]), have been associated with an increase in children’s BMI whereas healthier food environments (vendors/farmer’s market [14]) have been associated with decreases in BMI. Longitudinal studies regarding the influence of supermarkets have been mixed, with some finding supermarkets protect against weight gain in children [15] and others reporting increases in weight [18] or null results [7, 19]. To our knowledge, no studies have examined the longitudinal association between availability of food stores and BMI among Mexican children, who are exposed to a food environment mostly characterized by traditional retailers (small food stores and specialty stores) and an expansion driven by increases in convenience stores and supermarkets [20].

Understanding whether changes in the food environment are associated with changes in overweight and obesity prevalence in children is relevant because food environments could represent an additional opportunity for public policy interventions to promote healthier behaviors. Evidence from a systematic review of high-income countries support that the presence of retailers with healthy options, like fruit and vegetables may support modest-short term increases in fruit and vegetable consumption [21]. To fill these gaps, we aimed to estimate the association between changes in the retail food stores and the change in BMI in Mexican children and adolescents from 2002 to 2012. We hypothesized that increases in the density of fruit and vegetable stores and specialty food stores will be associated with a decrease in children and adolescent’s BMI. We hypothesized this because in Latin America, traditional specialized stores tend to sell unprocessed foods which are generally considered healthy (such as fruits, and vegetables, meat, fish and poultry, seeds, and legumes [22–24]). Additionally, we hypothesized that increases in the density of supermarkets, convenience stores and small food retail stores (*abarrotes* in Mexico) will be associated with an increase in BMI, considering that supermarkets and convenience stores in Latin America sell large volume of processed and ultra-processed foods and [22–24] Mexican households tend to purchase sugar-sweetened beverages in small food retail stores [25] and purchase ultra-processed food in supermarkets [26].

## Methods

### Study sample

**Fig. 1:**
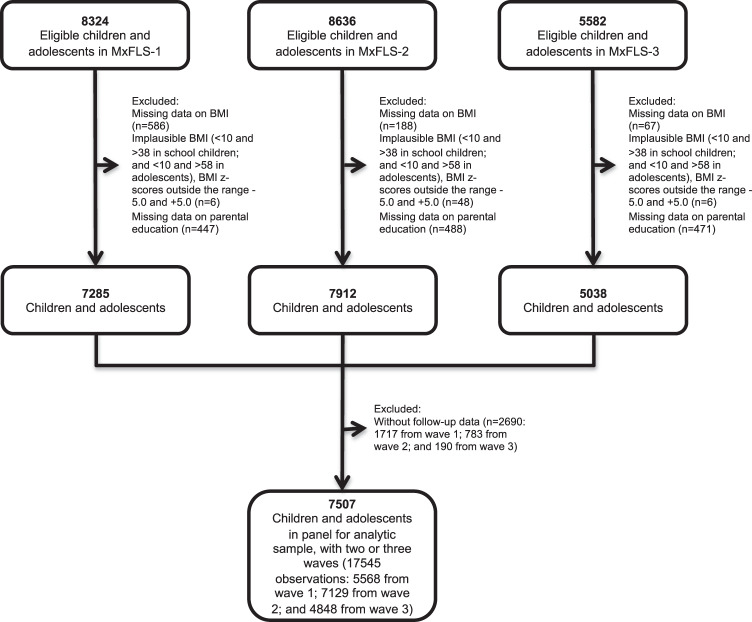
Flowchart of the study sample. MxFLS-1 the Mexican Family Life Survey wave 1 (2002); MxFLS-2 the Mexican Family Life Survey wave 2 (2005–2006); MxFLS-3 the Mexican Family Life Survey wave 3 (2009–2012); BMI Body mass index.

We analyzed anthropometric and sociodemographic data in school-aged children (5–11 years), and adolescents (12–19 years) from a longitudinal study with 10 years of follow-up. Individual-level panel data were extracted from the Mexican Family Life Survey (MxFLS), a longitudinal, multi-thematic survey representative of the Mexican population at the national, urban, rural, and regional level. MxFLS includes sociodemographic data and related health and nutritional measures. The first wave was conducted in 2002 (MxFLS-1) and recruited over 35000 individuals in 8440 households from 150 localities in Mexico. The study has 2 follow-ups, from 2005 to 2006 (MxFLS-2) and from 2009 to 2012 (MxFLS-3). Details of the MxFLS are available elsewhere [27–29]. For our analysis, the study population was limited to children aged 5–19 years during the three waves of the MxFLS who have complete information on age, sex, weight, height, and parental education level and had at least one follow-up (*n* = 7505, Fig. 1). We observed that the participants included in the complete follow-up were younger, had lower BMI, lower parental education, and a higher proportion of households located in very low deprivation areas than those with incomplete follow data (see Supplementary Table S1). A total of 174 (2.4% of the sample) children moved across municipalities during the follow-up period. This study was based on a secondary analysis of MxFLS data; the original protocol has the approval of the Ethics and Research Committee of the National Institute of Public Health of Mexico, and the National Institute of Perinatology of Mexico. All participants signed an informed consent and assent letter.

### Body mass index

Our primary outcome was the body mass index (BMI) of children aged 5–11 years and adolescents aged 12–19 years. Weight and height measures were obtained by trained personnel from the three waves of MxFLS survey. Weight was measured with a digital scale (Tanita) of the nearest 0.1 kg and height was measured to the nearest 0.1 cm with a SECA stadiometer [30]. Following previous research [31], we excluded from the analysis implausible BMI values: <10 kg/m^2^ and >38 kg/m^2^ for children, and <10 kg/m^2^ and >58 kg/m^2^ for adolescents. Standardized BMI-for-age z-score is widely used to assess childhood obesity [32], however, z-scores were developed on the basis of a cross-sectional reference and are inadequate to analyze longitudinal growth [33]. Therefore, we used unstandardized BMI values and controlled for age because this method is more sensitive to within-person change [34, 35].

### Retail food stores data

Our primary exposure of interest was the density of food stores by type for each municipality and year, obtained from the Economic Censuses from 1999, 2004 and 2009 (Automated Census Information System, *SAIC*) [36]. Data from the Economic Censuses were collected by the National Institute of Statistics and Geography of Mexico (*INEGI*) and contain information on the principal activity and addresses of establishments that carry out economic activities related to manufacturing, commerce and services [37]. The INEGI database includes the North American Industry Classification System (NAICS) to identify types of establishments. Detailed information about data collection is available elsewhere [37]. INEGI follows a systematized process to ensure the confidentiality of the data [38]; thus, the 1999, 2004 and 2009 Economic Censuses datasets had missing values in the count of some types of food stores for some municipalities. To inform these missing data points we conducted an imputation procedure described in Supplementary Document S1.

We categorized food stores according to their NAICS code in five mutually exclusive types, based on prior studies [39, 40]: (1) small food retail stores (small neighborhood corner stores, *tiendas de abarrotes* in Mexico), (2) specialty food stores (i.e., poultry market, meat market, fish/seafood market, dairy market, seed-grain stores, bakery), (3) fruit and vegetable stores, (4) convenience stores (chain convenience stores like OXXO, 7-Eleven, or local minimarkets), and (5) supermarkets (i.e., Walmart, Costco). The definitions, NAICS codes and examples of the food stores categories are available in Supplementary Table S2.

Each establishment had a municipal-level geo-identifier. We calculated the density of food stores within each municipality by dividing the number of food outlets by the total area of the municipality in km^2^ (boundaries were from 2000 INEGI’s Geostatistical framework [41]). Because many of the food density variables were right skewed and may have a non-linear association with BMI, we classified the density into quartiles for each food store type. Given the low numbers (and lower changes) of supermarkets in non-urban areas, we only created three categories of supermarket density for non-urban areas. Last, we also created a variable representing the proportion of food stores (number of each food store category over the total number of food stores) by type for each municipality and year.

### Linking children/adolescents to food store density

Each participant record in the three waves of the MxFLS had a municipal-level geo-identifier which changed if participants moved across municipalities. This municipal geo-identifier was linked to the municipal-level identifier in INEGI so that each participant record could be assigned the food store densities for their municipality.

### Covariates

Covariates were selected using Directed Acyclic Graphs and variables were included from the minimal sufficient adjustment sets for estimating the total effect of food stores on BMI (see Supplementary Fig. S1). We adjusted for participant sex (male or female) and age (in years). BMI is known to change non-linearly with age in children and adolescents, thus polynomials for age were included (linear, quadratic, and cubic age terms) [42, 43]. We also adjusted for parental education, a known factor for childhood obesity that is also correlated with area-level characteristics. Parental education represented the highest achieved level by either parent (five categories: no education, elementary, middle school, high school, more than high school). We also adjusted for two municipality-level variables: population density (using data from the National Census [44]) and a proxy of socioeconomic deprivation called the “marginality index” which was derived by the National Population Council [45]. We adjusted for these two municipality-level variables because they are known to be important contextual factors on BMI and the food environment [20, 46], being commonly adjusted [15, 16, 19]. The marginality index is a composite index of area-level socioeconomic deprivation that includes nine variables across four dimensions: access to public services, access to education, and economic and employment conditions. The index is divided into quintiles (quintile cut-points were based on the distribution for all the municipalities in Mexico) which correspond to: (1) very low, (2) low, (3) medium, (4) high and (5) very high deprivation [45]. For the purpose of our analysis, high and very high deprivation were collapsed into one category due to no observations (0%) in the very high deprivation category in non-urban areas. Both the socioeconomic deprivation and population density were calculated for years 2000, 2005 and 2010, and assigned to MxFLS records for 2002, 2005–06, 2009–12, respectively.

### Urbanization

The analyses were stratified by whether the municipality belongs to a city of at least 100,000 residents (as defined in the SALURBAL study [47, 48]) because availability of food retail differs by city size/urbanicity [49] and dietary behavior and health differ by city size/urbanicity [50].

### Statistical analysis

We described the data at baseline and for each year of follow-up. We computed means and standard deviations of continuous variables and proportions for categorical variables. We described the exposure variable (food stores) by the count of each food store type, the density of each food store type, and the percentage of food stores share by year.

To estimate the association between within-person change in exposure to municipality-level density of food stores and within-person change in BMI, we estimated five separate linear fixed-effects regression models, one per type of food store. The advantage of a fixed-effects approach is that time-invariant person and contextual-level characteristics are fully controlled for [51]. The model is specified, as follows:

$$\begin{array}{l} BMI_{ijt} = \beta _0 + \beta _1 \ast {{{\boldsymbol{Food}}}}\;{{{\boldsymbol{stores}}}}_{{{{\boldsymbol{it}}}}} + \beta _2 \ast Age_{it}\\ \qquad \qquad \,+ \,\beta _3 \ast Age_{it}^2 + \beta _4 \ast Age_{it}^3 + \beta _5 \ast {{{\boldsymbol{Parental}}}}\;{{{\boldsymbol{education}}}}_{{{{\boldsymbol{it}}}}}\\ \qquad \qquad \,+\, \beta _6 \ast Population\;density_{it} + \beta _7 \ast {{{\boldsymbol{Deprivation}}}}_{{{{\boldsymbol{it}}}}} + \alpha _i + \varepsilon _{it}\end{array}$$

Where the outcome variable is the BMI of subject *i* in municipality *j* at time *t* (numbers corresponding to the three MxFLS waves 2002, 2005–06, 2009–12); *α*_*i*_ is the subject-specific fixed effect; and *ε*_*it*_ is the error term for subject *i* at time *t*. The main exposure variable was food store density in four categories as defined by the quartiles of each type of food store; the lowest density quartile was the reference group. The coefficient β_1_ represents the change in BMI associated with an increase in each quartile of food stores compared to the reference (quartile 1), adjusted for the following time-varying variables: age, parental education level, municipal-level population density, and socioeconomic deprivation. We also tested whether there was a dose-response association by incorporating quartiles of food store density as an ordinal variable. Additionally, as a secondary analysis, we ran the set of models above using change in proportion of food stores by type (to proxy changes in the composition of the food environment) as the main exposure.

BMI is known to change non-linearly with age, thus polynomials for age were included according to improvements in Akaike’s information criterion (age centered at 5 years which was the youngest age observed in the cohort, adding quadratic and cubic terms) [42, 43]. To test whether BMI trajectories varied by quartiles of food stores, we added an interaction term between age (linear, squared, and cubed) and the categorical variable for food stores. The model is described in Supplementary Fig. S2. All analyses were conducted in Stata 14 (StataCorp, College Station, TX).

## Results

Table 1 presents participant characteristics overall and by urbanization. Participants were equally split between urban and non-urban areas. At baseline, participant mean age was 10 years, and 51.2% were women. The combined proportion of overweight and obesity increased over the 10-year period, from 30.0% to 36.6%, being higher in urban areas (urban areas 32.7% to 38.5%, non-urban areas 27.1% to 34.4%). Most parents completed elementary school, followed by middle school, with higher educational attainment in urban areas. Most children lived in the least deprived municipalities but municipalities in non-urban areas had higher levels of deprivation.

**Table 1:** Characteristics of participants at baseline and follow-up examination stratified by urbanization.

	Overall			Non-urban areas^a^			Urban areas^a^		
	Examination^b^			Examination^b^			Examination^b^		
	1 (*n* = 5568)	2 (*n* = 7129)	3 (*n* = 4848)	1 (*n* = 2601)	2 (*n* = 3349)	3 (*n* = 2315)	1 (*n* = 2967)	2 (*n* = 3780)	3 (*n* = 2533)
Age in years, [mean (SD)]	10.0 (3.0)	11.9 (3.8)	14.2 (2.8)	9.9 (3.0)	11.9 (3.8)	14.2 (2.8)	10.0 (3.0)	12.0 (3.9)	14.2 (2.9)
Age category, [%]									
School age (5–11 years)	71.1	50.0	25.4	72.1	50.9	25.2	70.2	49.3	25.5
Adolescents (12–19 years)	28.9	50.0	74.6	27.9	49.1	74.8	29.8	50.7	74.5
Sex, [%]									
Male	48.8	49.3	50.3	49.6	50.0	51.3	48.1	48.7	49.4
Female	51.2	50.7	49.7	50.4	50.0	48.7	51.9	51.3	50.6
Nutritional status									
Body mass index (kg/m^2^), [mean (SD)]	18.4 (3.7)	19.9 (4.4)	21.6 (4.4)	18.1 (3.5)	19.7 (4.3)	21.4 (4.4)	18.7 (3.8)	20.1 (4.4)	21.7 (4.5)
Body mass index category, [%]^c^									
Normal	67.9	62.8	61.8	70.8	65.1	63.9	65.4	60.7	59.8
Overweight (>+1 SD)	19.9	21.3	23.2	18.7	20.6	21.9	21.0	22.0	24.4
Obesity (>+2 SD)	10.1	12.9	13.4	8.4	11.7	12.5	11.7	14.0	14.1
Parental education level, [%]									
No education	4.0	3.2	2.1	6.0	5.1	3.2	2.3	1.5	1.1
Elementary	44.5	41.1	36.8	56.8	54	49.2	33.6	29.7	25.3
Middle school	29.3	30.6	32.3	23.8	25.5	29.2	34.2	35.1	35.1
High school	11.6	13.2	15.2	5.8	6.7	8.6	16.7	19.0	21.3
More than high school	10.6	12	13.6	7.7	8.7	9.7	13.2	14.8	17.2
Socioeconomic deprivation, [%]^d^									
Very low	41.2	42.2	44.3	9.3	12.6	13.7	69.2	68.5	72.2
Low	22.5	24.8	17.3	25.1	26.0	14.3	20.3	23.8	20.1
Medium	19.4	16.5	27.9	34.6	29.5	49.9	6.1	5.1	7.8
High-very high	16.9	16.4	10.6	31.0	31.9	22.1	4.4	2.6	0.0

Mexican Family Life Survey (MxFLS), 2002–2012.

*n* Sample size, *SD* standard deviation.

^a^Urbanization is defined by the population in 2010: urban areas are municipalities that belong to a city with more than 100,000 residents as defined by SALURBAL [47, 48], while non-urban areas refer to all other municipalities (see Methods section).

^b^Data from the Mexican Family Life Survey (MxFLS): Examination 1 = 2002; Examination 2 = 2005–2006; Examination 3 = 2009–2012.

^c^Calculated with BMI z-scores based on age and sex criteria from the WHO references of 2007.

^d^Data from the National Population Council (CONAPO): Examination 1 = 2000; Examination 2 = 2005; Examination 3 = 2010.

Table 2 shows the trends in the median number, density, and proportion of food stores by type and year. At baseline, small food retail and specialty stores dominated the food environment, followed by fruit and vegetable stores, convenience stores, and supermarkets. The density of food stores was higher in urban areas. Between 1999 and 2009 there was an increase in the median density of small food retail and specialty stores. Compared to non-urban areas, urban areas showed steeper changes in the median density of food stores, for instance, urban areas showed increases in the specialty and fruit and vegetable stores, and in non-urban areas all food stores stayed at similar levels, except for small food retail stores, which increased slightly. The range (min-max) of quartiles of food store densities and the distribution by urban/non-urban areas are presented in Supplementary Tables S3 and S4.

**Table 2:** Distribution of food stores in municipalities stratified by urbanization.

	Overall			Non-urban areas^a^			Urban areas^a^		
	1999 (*n* = 133)	2004 (*n* = 144)	2009 (*n* = 176)	1999 (*n* = 71)	2004 (*n* = 74)	2009 (*n* = 91)	1999 (*n* = 62)	2004 (*n* = 70)	2009 (*n* = 85)
	Median (p25, p75)	Median (p25, p75)	Median (p25, p75)	Median (p25, p75)	Median (p25, p75)	Median (p25, p75)	Median (p25, p75)	Median (p25, p75)	Median (p25, p75)
Median counts of food stores									
Small food retail stores	301 (92, 1105)	331 (95, 1389)	396 (118, 1180)	124 (41, 250)	114 (39, 268)	130 (42, 298)	1394 (404, 2331)	1415 (501, 2493)	1170 (517, 2659)
Specialty food stores	53 (12, 204)	54 (13, 280)	56 (15, 222)	18 (5, 34)	18 (3, 34)	18 (5, 48)	257 (77, 590)	280 (86, 570)	215 (75, 563)
Fruit and vegetable stores	25 (7, 82)	21 (6, 125)	25 (5, 102)	8 (2, 20)	7 (2, 16)	6 (2, 19)	106 (32, 413)	126 (38, 406)	83 (38, 404)
Convenience stores	2 (0, 12)	2 (0, 17)	2 (2, 29)	2 (0, 4)	2 (0, 5)	2 (2, 5)	6 (0, 71)	14 (2, 82)	24 (2, 107)
Supermarkets	0 (0, 3)	2 (0, 6)	2 (0, 7)	0 (0, 2)	0 (0, 2)	0 (0, 2)	2 (0, 11)	3 (2, 13)	6 (2, 18)
Median density of food stores									
Small food retail stores	0.4 (0.1, 1.5)	0.5 (0.1, 2.8)	0.6 (0.1, 3.3)	0.1 (0.0, 0.3)	0.1 (0.0, 0.4)	0.2 (0.0, 0.5)	2.3 (0.7, 9.4)	3.0 (0.8, 13.9)	3.3 (0.8, 14.4)
Specialty food stores	0.1 (0.0, 0.3)	0.1 (0.0, 0.5)	0.1 (0.0, 0.6)	0.0 (0.0, 0.1)	0.0 (0.0, 0.1)	0.0 (0.0, 0.1)	0.4 (0.1, 2.6)	0.5 (0.1, 2.8)	0.5 (0.1, 2.9)
Fruit and vegetable stores	0.0 (0.0, 0.2)	0.0 (0.0, 0.3)	0.0 (0.0, 0.3)	0.0 (0.0, 0.0)	0.0 (0.0, 0.0)	0.0 (0.0, 0.0)	0.2 (0.1, 1.2)	0.3 (0.1, 2.3)	0.3 (0.1, 1.1)
Convenience stores	0.0 (0.0, 0.0)	0.0 (0.0, 0.0)	0.0 (0.0, 0.0)	0.0 (0.0, 0.0)	0.0 (0.0, 0.0)	0.0 (0.0, 0.0)	0.0 (0.0, 0.1)	0.0 (0.0, 0.1)	0.0 (0.0, 0.2)
Supermarkets	0.0 (0.0, 0.0)	0.0 (0.0, 0.0)	0.0 (0.0, 0.0)	0.0 (0.0, 0.0)	0.0 (0.0, 0.0)	0.0 (0.0, 0.0)	0.0 (0.0, 0.0)	0.0 (0.0, 0.0)	0.0 (0.0, 0.0)
Median proportion of food stores									
Small food retail stores	75.2 (67.3, 84.0)	76.8 (69.4, 84.1)	76.2 (68.3, 81.5)	78.3 (69.9, 86.6)	80.84 (71.2, 87.2)	77.1 (66.8, 85.4)	74.0 (65.2, 79.1)	73.9 (67.6, 79.6)	74.0 (69.5, 79.6)
Specialty food stores	14.4 (8.6, 19.8)	13.9 (7.9, 18.4)	14.5 (8.1, 18.0)	11.9 (6.7, 18.0)	11.57 (6.1, 17.6)	13.1 (6.3, 18.0)	15.6 (11.7, 21.2)	14.7 (11.3, 20.7)	14.7 (11.1, 18.0)
Fruit and vegetable stores	7.1 (3.1, 11.5)	5.6 (2.6, 10.4)	6.2 (2.9, 10.5)	5.9 (2.3, 11.1)	3.80 (1.0, 8.8)	4.5 (2.0, 10.2)	8.3 (4.5, 12.1)	6.9 (3.8, 12.3)	7.1 (3.8, 10.7)
Convenience stores	0.4 (0.0, 2.8)	0.4 (0.0, 3.0)	1.2 (0.2, 3.9)	0.4 (0.0, 2.5)	0.33 (0.0, 2.5)	1.1 (0.1, 3.3)	0.4 (0.0, 3.7)	0.8 (0.1, 3.7)	1.3 (0.3, 4.0)
Supermarkets	0.0 (0.0, 0.3)	0.1 (0.0, 0.4)	0.1 (0.0, 0.4)	0.0 (0.0, 0.1)	0.00 (0.0, 0.3)	0.0 (0.0, 0.3)	0.1 (0.0, 0.4)	0.2 (0.0, 0.4)	0.3 (0.1, 0.5)

Economic Census 1999, 2004 and 2009.

*n* observations of municipalities, *p25* 25th percentile, *p75* 75th percentile.

^a^Urbanization is defined by the population in 2010: urban areas are municipalities that belong to a city with more than 100000 residents as defined by SALURBAL [47, 48], while non-urban areas refer to all other municipalities (see Methods section).

Table 3 shows the overall adjusted association between changes in food store density for each type of store and within-person differences in BMI (urban and non-urban areas combined). There was an inverse dose-response association between increases in the density of specialty food stores, and fruit and vegetable stores and BMI. This trend was statistically significant for fruit and vegetable store density (*P* < 0.001) but not for other food store types. For example, there was a decrease of 0.455 kg/m^2^, 0.733 kg/m^2^, and 0.838 kg/m^2^ in BMI in the second, third, and fourth quartile of changes in fruit and vegetable store density, as compared to the first quartile. Results also suggested a positive association between increases in the density of convenience stores and supermarkets and increases in BMI. Trends in Table 3 were not monotonic, and only the statistically significant p-trend for supermarkets suggest an association (*P* < 0.006).

**Table 3:** Within-person mean difference in body mass index associated with within-person mean differences in quartile of food stores.

	Model^a^		
	β	95% CI	*P*-value
Density of small food retail stores			0.893^b^
Quartile 1 (0.0004–0.14)	Ref.		
Quartile 2 (0.15–0.556)	−0.110	−0.398, 0.178	0.455
Quartile 3 (0.561–2.51)	−0.068	−0.446, 0.310	0.723
Quartile 4 (2.58–91.46)	0.090	−0.449, 0.629	0.743
Density of specialty food stores			0.453^b^
Quartile 1 (0–0.013)	Ref.		
Quartile 2 (0.014–0.078)	−0.126	−0.400, 0.149	0.369
Quartile 3 (0.08–0.36)	−0.201	−0.601, 0.198	0.323
Quartile 4 (0.39–67.08)	−0.089	−0.697, 0.518	0.774
Density of fruit and vegetable stores			**<0.001**^b^
Quartile 1 (0–0.0061)	Ref.		
Quartile 2 (0.0062–0.0402)	−0.455	−0.704, −0.207	**<0.001**
Quartile 3 (0.0404–0.22)	−0.733	−1.072, −0.394	**<0.001**
Quartile 4 (0.23–129.09)	−0.838	−1.291, −0.385	**<0.001**
Density of convenience stores			0.459^b^
Quartile 1 (0–0.0001)	Ref.		
Quartile 2 (0.0002–0.006)	0.035	−0.178, 0.247	0.750
Quartile 3 (0.01–0.0398)	0.059	−0.118, 0.236	0.515
Quartile 4 (0.040–8.02)	0.077	−0.168, 0.322	0.536
Density of supermarkets			**0.006**^b^
Quartile 1 (0)	Ref.		
Quartile 2 (0.0001–0.0009)	−0.068	−0.380, 0.243	0.666
Quartile 3 (0.001–0.009)	0.310	0.145, 0.476	**<0.001**
Quartile 4 (0.01–1.23)	0.164	−0.074, 0.403	0.177

Mexican Family Life Survey (MxFLS), 2002–2012 and Economic Census 1999, 2004 and 2009.

Number of observations = 17545; number of participants = 7507.

*β* Coefficients of the regressors, *95% CI* 95% Confidence Interval.

^a^Five linear fixed-effects regression models (one per food store type) adjusted for age, age^2^, age^3^, parental education level, population density, and socioeconomic deprivation.

^b^The *p*-value for trend comes from a model with quartiles as an ordinal variable.

*P*-values <0.05 were considered significant and are highlighted in bold.

When exposures were operationalized as the change in proportion of food stores (number of each food store category over the total number of food stores), results were only statistically significant for proportion of supermarkets (Supplementary Table S5). For example, for each ten unit increase in the percent of supermarkets (out of all food stores), BMI increased on average 1.322 kg/m^2^ (95% CI: 0.139, 2.506); in stratified analyses, this association was only observed in non-urban areas.

Table 4 shows the adjusted association between change in food store density and change in BMI for urban and non-urban areas. After stratification, the inverse association between increases in the density of fruit and vegetable stores and decrease of BMI was only significant for non-urban municipalities. Whereas the inverse association between increases in the density of specialty food stores and decrease of BMI was only significant for urban municipalities. Further, there was a positive association between the largest increase in the density of supermarkets and higher BMI in non-urban areas.

**Table 4:** Within-person mean difference in body mass index associated with within-person mean differences in quartile of food stores, stratified by urbanization.

Model^a^							
Non-urban areas^b^				Urban areas^b^			
	β	95% CI	*P*-value		β	95% CI	*P*-value
Density of small food retail stores			**0.031**^c^	Density of small food retail stores			0.698^**c**^
Quartile 1 (0.0004–0.040)	Ref.			Quartile 1 (0.06–0.6087)	Ref.		
Quartile 2 (0.041–0.17)	−0.083	−0.540, 0.374	0.722	Quartile 2 (0.6095–2.07)	0.038	−0.269, 0.345	0.810
Quartile 3 (0.19–0.36)	−0.573	−1.209, 0.064	0.078	Quartile 3 (2.25–6.09)	−0.525	−1.123, 0.072	0.085
Quartile 4 (0.39–3.70)	−0.840	−1.612, −0.068	**0.033**	Quartile 4 (6.15–91.46)	−0.058	−0.943, 0.827	0.897
Density of specialty food stores			0.557^**c**^	Density of specialty food stores			**0.002**^c^
Quartile 1 (0–0.0040)	Ref.			Quartile 1 (0.01–0.0731)	Ref.		
Quartile 2 (0.0041–0.0215)	0.184	−0.186, 0.554	0.329	Quartile 2 (0.0734–0.327)	−0.323	−0.932, 0.286	0.299
Quartile 3 (0.0220–0.071)	0.458	−0.043, 0.959	0.073	Quartile 3 (0.335–1.38)	−0.789	−1.512, −0.066	**0.033**
Quartile 4 (0.074–1.11)	0.249	−0.333, 0.831	0.402	Quartile 4 (1.42–67.08)	−1.204	−2.046, −0.362	**0.005**
Density of fruit and vegetable stores			**0.007**^c^	Density of fruit and vegetable stores			0.936^**c**^
Quartile 1 (0–0.0010)	Ref.			Quartile 1 (0–0.028)	Ref.		
Quartile 2 (0.0010–0.0078)	−0.265	−0.515, −0.015	**0.038**	Quartile 2 (0.031–0.200)	0.156	−0.415, 0.726	0.593
Quartile 3 (0.008–0.04)	−0.079	−0.547, 0.389	0.741	Quartile 3 (0.204–0.77)	0.270	−0.441, 0.981	0.457
Quartile 4 (0.05–0.55)	−0.487	−1.042, 0.068	0.085	Quartile 4 (0.81–129.09)	0.092	−0.709, 0.894	0.821
Density of convenience stores			0.731^**c**^	Density of convenience stores			0.731^**c**^
Quartile 1 (0)	Ref.			Quartile 1 (0–0.0055)	Ref.		
Quartile 2 (0.0001–0.0013)	0.365	−0.174, 0.903	0.185	Quartile 2 (0.006–0.0333)	0.134	−0.106, 0.375	0.273
Quartile 3 (0.0013–0.0057)	−0.151	−0.402, 0.100	0.240	Quartile 3 (0.0340–0.1031)	0.153	−0.179, 0.486	0.365
Quartile 4 (0.006–0.14)	0.025	−0.225, 0.275	0.843	Quartile 4 (0.1033–8.02)	0.047	−0.283, 0.377	0.779
Density of supermarkets^d^			**0.042**^c^	Density of supermarkets			0.420^**c**^
Quartile 1 (0)	Ref.			Quartile 1 (0–0.0011)	Ref.		
Quartile 2				Quartile 2 (0.0011–0.0078)	0.213	−0.024, 0.451	0.079
Quartile 3 (0.0001–0.0005)	−0.357	−0.821, 0.108	0.132	Quartile 3 (0.0083–0.023)	0.212	−0.089, 0.514	0.168
Quartile 4 (0.0006–0.02)	0.283	0.049, 0.517	**0.018**	Quartile 4 (0.024–1.23)	0.068	−0.311, 0.447	0.725

Mexican Family Life Survey (MxFLS), 2002–2012 and Economic Census 1999, 2004 and 2009.

Non-urban areas: Number of observations = 8265; number of participants = 3544. Urban areas: Number of observations = 9280; number of participants = 4049.

*β* Coefficients of the regressors, *95% CI* 95% Confidence Interval.

^a^Five linear fixed-effects regression models (one per food store type) adjusted for age, age^2^, age^3^, parental education level, population density, and socioeconomic deprivation.

^b^Urbanization is defined by the population in 2010: urban areas are municipalities that belong to a city with more than 100,000 residents as defined by SALURBAL [47, 48], while non-urban areas refer to all other municipalities (see Methods section).

^c^The *p*-value for trend comes from a model with quartiles as an ordinal variable.

^d^Due to distribution of density of supermarkets, the second quartile was not able to be estimated.

*P*-values <0.05 were considered significant and are highlighted in bold.

Supplementary Tables S6a–S6e show the marginal effects of changes in food store density across different age groups. On average, the association between changes in food store density by type and changes in BMI differed by age (*p* for interaction <0.001). For example, on average, living in municipalities with a higher density of fruit and vegetable stores (across all quartiles of fruit/vegetable stores) was associated with a decreasing trend of BMI for all ages, but the magnitude was higher for adolescents (see Supplementary Table S6c). An increase in the density of convenience stores was associated with increases in BMI in participants aged 9 to 13, but with BMI reductions among those aged 17–19 (see Supplementary Table S6d). The highest quartile of density of supermarkets was associated with an increasing trend of BMI in earlier ages, but a decrease in BMI for later years (see Supplementary Table S6e).

## Discussion

Using longitudinal data from children and adolescents in Mexico, results suggested an inverse dose-response association between increases in the density of small food retail stores, specialty food stores, and fruit and vegetable stores and a reduction in BMI. In contrast, increases in the density of convenience stores and supermarkets were associated to increases in BMI. Our findings show that children living in areas with increasing density of fruit and vegetable stores have declines (or slower increases) in BMI during childhood and adolescence, especially in non-urban areas. Children living in urban areas showed an inverse association between changes in specialty food store density and BMI. Further, results suggested stronger responses in adolescents, with changes in the density of fruit and vegetable stores being more strongly associated with lower BMI at older ages.

The longitudinal evidence on the association between food store types and BMI is scarce, mostly related to high income countries, focused on short follow-up periods in childhood (<10 years of follow-up), and with mixed results [14–16, 18, 19]. For example, one study from California found an inverse association between fruit and vegetable stores and farmer’s market and the risk of overweight/obesity [14], whereas another study found a positive association [18]. Differences in the age groups of these studies may account for some of this discordance (aged 6–7 in the former and aged 3–5 in the later) [14, 18]. Indeed, our study found stronger associations for older ages, which suggests that age could be an important effect modifier in the association between the food environment and BMI (Supplementary Table S6c). The inverse association in this study may be related to food purchases in stores, an increase in the diet quality of children, or a higher availability of fruit/vegetables at home. A new study from Mexico, algins with our study finding density of fruit/vegetable stores are associated with higher purchases of fruit and vegetables [26]. Also, prior studies in children and adolescents showed that the availability of fruit/vegetable stores in residential/school environment [52, 53], or home environment [54] was associated with higher weight-related behaviors like the fruit and vegetable consumption.

We did not observe a consistent association between supermarket density and BMI. This is similar to longitudinal studies among children in USA that found null associations between availability of supermarkets and BMI [14, 17], as compared to other studies that showed inverse associations [15]. These differences across studies may be related to contextual differences in the food environment, including the type of products that supermarkets carry. For instance, in high income countries, studies have shown that, although supermarkets have the highest healthy food availability [55, 56], they also procure unhealthy ultra-processed foods [23, 57]. In Latin America, the evidence has highlighted the role of supermarkets in providing access to unhealthy foods [22, 24]. In Mexican supermarkets, current in-store marketing practices, like placement, shelf space and price promotions, are predominantly promoting unhealthy products targeting children [58]. Recent studies in Mexico showed a direct association between density of supermarkets with sugar-sweetened beverages consumption and with overweight/obesity [12], as well as with higher purchases of ultra-processed food [26]. Unfortunately, the context of the consumer food environment in Mexico is still understudied.

We also found inverse associations between specialty stores (i.e., poultry market, meat market, fish/seafood market, dairy market, seed-grain stores, bakery) and BMI exclusively in urban areas. In developed countries, specialized stores generally source unprocessed foods, with little to no presence of processed and ultra-processed food [56, 57]. Evidence from Mexican households suggest that density of stores specialized in selling animal based-food are inversely associated with purchases of ultra-processed food and sugar-sweetened beverages, and positively associated with higher purchases of fruit and vegetable stores in urban areas [26]. Evidence point at the role of sugar-sweetened beverages [3] and ultra-processed food [59] in BMI increase (or overweight/obesity), thus, access to specialized stores could increase availability of unprocessed food, leading to lower BMI.

Interestingly, our results also suggest a dynamic association between the food environment and BMI that varies by age, with adolescents having a stronger association. This may be related to differences between children and adolescents in how they interact with their food environment, as previous research has hypothesized that older children develop more autonomy and interact more with their environment [60]. Adolescent girls have been shown to be independent in determining their food intake away from home [61]. Another study suggests that early adolescent children (10–14 years) experience independence and mobility in their local food environment, and that they tend to purchase almost exclusively energy dense snacks from convenience stores, gas stations and grocery stores [62].

The results of our study highlight the importance of the food environment as a contextual factor of nutritional outcomes in children and adolescents. They underline the importance of healthy food stores, especially in non-urban areas. In terms of public policies, the evidence generated through our study emphasizes the importance of certain store types such as fruit and vegetable, and specialty stores where children and/or their parents may access healthier foods. Promoting these stores should be included within strategies aimed at improving the nutritional health of the Mexican child population. For instance, interventions to improve the location and density of retailers selling healthy food [21]. Due to the scientific rigor of our study, its results can support evidence-based policies.

Despite the strengths of the longitudinal data and study design used, our study has some limitations. First, the study has a 10 year follow-up, in which children transitioned from school-age to adolescence, and we lacked measurements on pubertal stages that could affect BMI [63]. However, we modeled BMI using multiple age polynomials to allow flexibility in the BMI growth trajectories. Second, selection bias is possible if parents selected their/child’s municipality based on availability of certain types of food stores. However, in our study there were very few children who moved across municipalities (*N* = 174, <3%), making our sample a “non-mover” sample and reducing the likelihood of selection bias (sensitivity analysis excluding children who moved had no impact on the estimates of our models, data not shown). Third, we evaluated the community food environment through the density of food stores, yet it is important to also evaluate the consumer food environment, which reflects what consumers encounter within stores, for instance, availability, variety, pricing, promotions, and nutritional quality of products [64]. Fourth, we did not consider the spatial or temporal accessibility to food stores [6], due to confidentiality on geographical location of households of the data, and computed city-level densities instead. Additionally, we could not include informal food markets which offer both healthy and unhealthy options -- such as temporary open-air street markets that are open one- or two days per week -- because there is no information available about these.

## Conclusion

In summary, we found that the distribution of the retail food environment in Mexico, especially the availability of fruit/vegetable stores, may influence children and adolescent BMI. The inverse association between fruit/vegetable stores, and specialty food stores with BMI are different across urbanization, pointing at the complexity of studying the retail food environment across the continuum of urbanization. Future studies are needed to further understand the mechanisms through which availability of retail food environment is influencing the change in BMI in children.

## Supplementary information

Supplementary material

## Data Availability

The present study is a secondary analysis of data from public information sources. The individual-level panel data from the Mexican Family Life Survey (MxFLS) is available at http://www.ennvih-mxfls.org/index.html. Area-level data of retail food stores from the Economic Censuses are publicly available at https://www.inegi.org.mx/app/saich/v1/?evt=1999 (1999), https://www.inegi.org.mx/app/saich/v1/?evt=2004 (2004) and https://www.inegi.org.mx/app/saich/v1/ (2009). Socioeconomic deprivation and population were obtained through the National Population Council available at https://datos.gob.mx/busca/dataset/indice-de-marginacion-carencias-poblacionales-por-localidad-municipio-y-entidad.

## Code availability

Available from the first author upon request.
