# Supplementary material for: Longitudinal association between density of retail food stores and body mass index in Mexican school children and adolescents
Source: Int J Obes (Lond). 2023 Feb 15;47(5):365–74. doi: 10.1038/s41366-023-01273-w (PMC10147568; doi:10.1038/s41366-023-01273-w)
Supplement: Supplementary file 1 — Supplementary material [file 41366_2023_1273_MOESM1_ESM.docx]

**SUPPLEMENTARY MATERIAL**

**File contents**

**Table S1.** Baseline characteristics of the participants from the case complete analysis as compared with those who lost to follow-up either in any of the follow-up waves.

**Document S1.** Imputation procedure of retail food environment data.

**Table S2.** Food store categories.

**Figure S1.** Directed Acyclic Graphs (DAGs).

**Figure S2.** Specification of fixed-effects linear regression model with interaction terms.

**Table S3.** Distribution of quartiles of food stores. Economic Census 1999, 2004 and 2009.

**Table S4.** Distribution of quartiles of food stores by urbanization. Economic Census 1999, 2004 and 2009.

**Table S5.** Within-person mean difference in body mass index associated with within-person mean differences in proportion of food stores.

**Table S6a.** Marginal effects considering interaction between age and quartiles of density of small food retail stores on body mass index trajectories.

**Table S6b.** Marginal effects considering interaction between age and quartiles of density of specialty food stores on body mass index trajectories.

**Table S6c.** Marginal effects considering interaction between age and quartiles of density of fruit and vegetable stores on body mass index trajectories.

**Table S6d.** Marginal effects considering interaction between age and quartiles of density of convenience stores on body mass index trajectories.

**Table S6e.** Marginal effects considering interaction between age and quartiles of density of supermarkets on body mass index trajectories.

|  | **Baseline (participants with complete data)** | | |
| --- | --- | --- | --- |
| **Characteristics** | **Complete follow-up** | **Lost to follow-up** | **p-value** |
|  | **n= 7507** | **n= 2690** |  |
| Age in years, [mean (SD)] | 9.4 (3.0) | 12.0 (3.6) | **<0.001** |
| Age category, [%] |  |  |  |
| School age (5-11 years) | 75.8 | 42.2 | **<0.001** |
| Adolescents (12-19 years) | 24.2 | 57.8 | **<0.001** |
| Sex, [%] |  |  |  |
| Male | 49.4 | 50.0 | 0.623 |
| Female | 50.6 | 50.0 | 0.623 |
| Nutritional status |  |  |  |
| Body mass index (kg/m^2^), [mean (SD)] | 18.2 (3.8) | 20.3 (4.7) | **<0.001** |
| Body mass index category, [%]^a^ |  |  |  |
| Normal | 66.7 | 63.2 | **0.001** |
| Overweight (>+1SD) | 19.1 | 21.1 | **0.027** |
| Obesity (>+2SD) | 11.8 | 13.8 | **0.007** |
| Parental education level, [%] |  |  |  |
| No education | 3.8 | 5.6 | **<0.001** |
| Elementary | 42.6 | 42.7 | 0.909 |
| Middle school | 30.4 | 27.3 | **0.002** |
| High school | 12.4 | 12.0 | 0.559 |
| More than high school | 10.8 | 12.4 | **0.02** |
| Socioeconomic deprivation, [%]^b^ |  |  |  |
| Very low | 41.5 | 51.3 | **<0.001** |
| Low | 23.0 | 20.6 | **0.011** |
| Medium | 18.4 | 14.2 | **<0.001** |
| High-very high | 17.1 | 13.9 | **<0.001** |
| n = Sample size; SD = standard deviation. | | | |
| ^a^ Calculated with BMI z-scores based on age and sex criteria from the WHO references of 2007. | | | |
| ^b^ Data from the National Population Council (CONAPO) | | | |

**Table S1. Baseline characteristics of the participants from the case complete analysis as compared with those who lost to follow-up either in any of the follow-up waves.**

**Document S1. Imputation procedure of retail food environment data.**

1. Review of dataset
2. The exposure variables were the food store types at each municipality and year. We used the Economic Censuses dataset of years 1999, 2004 and 2009 (this data was merge to the individual level data from MxFLS, which has three waves from 2002, 2005-2006 and 2009-2012).
3. The dataset was download from the *Sistema Automatizado de Información Censal (SAIC)* from INEGI:
4. Web page from 1999: <https://www.inegi.org.mx/app/saich/v1/?evt=1999>
5. Web page from 2004: <https://www.inegi.org.mx/app/saich/v1/?evt=2004>
6. Web page from 2009: <https://www.inegi.org.mx/app/saich/v1/>
7. The dataset had missing values in the count of each type of food stores, this due to confidentiality of the data. The number of economic units (counts by municipality), with a value equal to 1 or 2, are reserved under the principles of confidentiality established by law. An algorithm was defined that is focused on taking care of confidentiality of records that have 1 or 2 economic units through the use of another registry that works as an encrypted to keep the confidentiality and security of the information intact for all levels of geographic or sectoral disaggregation that results in these values. Once that the activity classes (from the economic units) have been identified with confidentiality, a criterion is applied that eliminates any possibility of obtaining the data by difference. See methodological document from 2014: <https://www.inegi.org.mx/contenidos/app/saic/saic_metodo.pdf>
8. Then, we explored the number and proportion of missing values per each food store type for 1999, 2004 and 2009. We restricted the municipalities to the MxFLS analytic sample (where is the outcome variable BMI at the individual-level):

| **Number and proportion of missing values for 1999. Municipalities from MxFLS sample** | | | |  | **Number and proportion of missing values for 2004. Municipalities from MxFLS sample** | | | |  | **Number and proportion of missing values for 2009. Municipalities from MxFLS sample** | | | |
| --- | --- | --- | --- | --- | --- | --- | --- | --- | --- | --- | --- | --- | --- |
| **Food store type*** | **# Missing** | **Total municipalities** | **% Missing** |  | **Food store type** | **# Missing** | **Total municipalities** | **% Missing** |  | **Food store type** | **# Missing** | **Total municipalities** | **% Missing** |
| **Grocery stores** | 16 | 133 | 12.03 |  | **Grocery stores** | 20 | 144 | 13.89 |  | **Grocery stores** | 21 | 176 | 11.93 |
| **Beverages stores** | 50 | 133 | 37.59 |  | **Beverages stores** | 49 | 144 | 34.03 |  | **Beverages stores** | 68 | 176 | 38.64 |
| **Meat stores** | 25 | 133 | 18.8 |  | **Meat stores** | 33 | 144 | 22.92 |  | **Meat stores** | 42 | 176 | 23.86 |
| **Poultry stores** | 43 | 133 | 32.33 |  | **Poultry stores** | 47 | 144 | 32.64 |  | **Poultry stores** | 54 | 176 | 30.68 |
| **Fish stores** | 32 | 133 | 24.06 |  | **Fish stores** | 40 | 144 | 27.78 |  | **Fish stores** | 43 | 176 | 24.43 |
| **Dairy stores** | 39 | 133 | 29.32 |  | **Dairy stores** | 46 | 144 | 31.94 |  | **Dairy stores** | 43 | 176 | 24.43 |
| **Seed and grain stores** | 40 | 133 | 30.08 |  | **Seed and grain stores** | 57 | 144 | 39.58 |  | **Seed and grain stores** | 37 | 176 | 21.02 |
| **Fruit and vegetable stores** | 19 | 133 | 14.29 |  | **Fruit and vegetable stores** | 22 | 144 | 15.28 |  | **Fruit and vegetable stores** | 25 | 176 | 14.2 |
| **Convenience stores** | 60 | 133 | 45.11 |  | **Convenience stores** | 76 | 144 | 52.78 |  | **Convenience stores** | 92 | 176 | 52.27 |
| **Supermarkets** | 36 | 133 | 27.07 |  | **Supermarkets** | 53 | 144 | 36.81 |  | **Supermarkets** | 58 | 176 | 32.95 |
| * Small food retail stores (includes Grocery stores= NAICS code 461110 - Retail trade in grocery stores; and Beverage stores= NAICS code 461213 - Retail trade of nonalcoholic beverages and ice); Specialty food stores (includes Meat stores = NAICS code 461121 - Retail trade of red meat; Poultry stores = NAICS code 461122 - Retail trade of poultry; Fish stores = NAICS code 461123 - Retail trade of fish and seafood; Dairy stores = NAICS code 461160 - Retail trade of milk, other dairy products and sausages; and Seed and grain stores = NAICS code 461150 - Retail trade of food seeds and grains, spices and dried chili); Fruit and vegetable stores (NAICS code 461130 - Retail trade of fresh fruits and vegetables); Convenience stores (NAICS code 462112 - Retail trade in minimarkets); Supermarkets (NAICS code 462111 - Retail trade in supermarkets) | | | | | | | | | | | | | |

1. Review of the encrypted data
2. We decided to explore better the encrypted data longitudinally (long format dataset from 1999, 2004 and 2009), because we realized that some data are not exclusive plausible to be <3 counts (as INEGI referred in methodological document.
3. Then, we made the differentiation of missing patterns (encrypted values) in a systematic way with rules. That is, generate a variable that identifies those missing data and within those missing data which are suspicious (which are not plausible values less than 3) by panel identifier, which is the municipality:
4. 1 – TRUE: When we are "sure" they are encrypted values according to INEGI (values less than 3):

- Identified those municipalities where the three waves have encrypted values (1999, 2004 and 2009).
- Identified those municipalities that have at list 1 encrypted data and at list 1 value less or equal than 3.

1. 2 – COMPLETE data: Identified those municipalities where the three waves have complete data.
2. 0 – FALSE: When we suspect that the encrypted values are not less than 3:

- Municipalities where there is at list a value greater than 3.

1. We identified the patterns of the “FALSE” category, those values that we are not sure they are encrypted values according to INEGI. So, in this way we can decide what to do with this data (interpolation or extrapolation).

Combinations or patterns:

| **FALSE Patterns** | | | | | | |
| --- | --- | --- | --- | --- | --- | --- |
|  | False – 1 missing in 2004 | False – 1 missing in 2009 | False – 1 missing in 1999 | False – 2 missing | False – 2 missing | False – 2 missing |
| **1999** | ✓ | ✓ | * | ✓ | * | * |
| **2004** | * | ✓ | ✓ | * | * | ✓ |
| **2009** | ✓ | * | ✓ | * | ✓ | * |
| **To do** | Interpolation | Extrapolation | Extrapolation | Does not apply | Does not apply | Does not apply |

1. For the “FALSE” patterns with 2 missing, it could be extrapolated or interpolated, considering the National Statistical Directory of Economic Units (DENUE) 2014 dataset (which has complete data). The DENUE is an inventory that contains information on the principal economic activity and location of economic units [1]. The information of DENUE is based on the National Economic Censuses. When the DENUE 2014 database is added, it is possible to interpolate/extrapolate even those municipalities that have two encrypted data. Since the extrapolation does is take at least two data to be able to estimate in the same way that linear interpolation is done.

Combinations of patters:

| **FALSE Patterns** | | | | | | |
| --- | --- | --- | --- | --- | --- | --- |
|  | False – 1 missing in 2004 | False – 1 missing in 2009 | False – 1 missing in 1999 | False – 2 missing | False – 2 missing | False – 2 missing |
| **1999** | ✓ | ✓ | * | ✓ | * | * |
| **2004** | * | ✓ | ✓ | * | * | ✓ |
| **2009** | ✓ | * | ✓ | * | ✓ | * |
| **2014** | ✓ | ✓ | ✓ | ✓ | ✓ | ✓ |
| **To do** | Interpolation | Extrapolation | Extrapolation | Interpolation | Extrapolation | Interpolation |

1. Data management
2. Imputation: Applied to the “TRUE” missing pattern. We imputed the 1.5 value for all those observations.
3. Linearly interpolate and extrapolate values: Applied to the “FALSE” missing pattern. We used a STATA command “ipolate”. Panel data should be interpolated separately -by <panel identifier>, in this case by municipality identifier. “ipolate” creates in newvar a linear interpolation of yvar on xvar for missing values of yvar [2].

The value y at x is found by finding the closest points (x_0_ , y_0_) and (x_1_, y_1_), such that x_0_ < x and x_1_ < x where y_0_ and y_1_ are observed, and calculating:

$$\mathcal{Y=}\frac{\mathcal{Y}_{1}-\mathcal{Y}_{0}}{\mathcal{X}_{1}-\mathcal{X}_{0}} \left( \mathcal{X-}\mathcal{X}_{0} \right)+\mathcal{Y}_{0}$$

| **Food store type** | **NAICS codes of the economic unit** | **Examples of food stores** |
| --- | --- | --- |
| Small food retail food stores (a*barrotes*) | 461110 - Retail trade in grocery stores + 461213 - Retail trade of nonalcoholic beverages and ice | Traditional stores that sell milk, cheese, cream, cold cuts, sweets, cookies, bread, cakes, snacks, fried foods, preserves, canned goods, bottled purified water, soft drinks, juices and nectars, hydrating drinks, energy drinks, beer, packaged wines and spirits, cigars, egg, toilet paper, detergent, soap, paper napkins, disposable kitchen utensils. No chain names available. |
| Specialty food stores | 461121 - Retail trade of red meat + 461122 - Retail trade of poultry + 461123 - Retail trade of fish and seafood + 461160 - Retail trade of milk, other dairy products and sausages^a^ + 461150 - Retail trade of food seeds and grains, spices and dried chili^a^ | Traditional stores that sells red meat (raw or semi-cooked beef, pork, lamb, goat and other species of red meat animals), poultry (poultry offal, chicken, quail, duck, turkey), fishmongers (fish or shellfish fresh, dried, salted and frozen, and other marine products), dairy and sausages (milk, cream, butter, yogurt, cheese, chorizo, sausage, mortadella, ham, pork cheese), and other food (coffee, bread, bakery, tortillas, eggs). These stores could be inside public food markets or not. No chain names available. |
| Fruit and vegetable stores | 461130 - Retail trade of fresh fruits and vegetables | Traditional stores mainly dedicated to the specialized retail trade of fresh fruits and vegetables. These stores could be inside public food markets or not. No chain names available. |
| Convenience stores | 462112 - Retail trade in minimarkets | Stores mainly dedicated to the retail trade of food, beverages, and basic necessities, organized in sections or small specialized exhibition areas that facilitate direct public access to merchandise (could be chain convenience stores like OXXO, 7-Eleven, Circle K, Extra, or local stores). |
| Supermarkets | 462111 - Retail trade in supermarkets | Wal-Mart, H-E-B, Soriana, Comercial Mexicana, Superama, City-Market, Fresko, Costco, Sam´s Club, among others. |
| Source: Own elaboration adapted from NAICS 1997, 2002, and 2007. The National Institute of Statistics and Geography (INEGI). North American Industry Classification System (NAICS) 2007. Mexico; 2007. | | |
| ^a^ Those NAICS codes change for Economic Census from 2009: 461150 - Retail trade of milk, other dairy products and sausages, and 461140 - Retail trade of food seeds and grains, spices and dried chili. | | |

**Table S2. Food store categories.**

**Figure S1. Directed Acyclic Graphs (DAGs)**

**
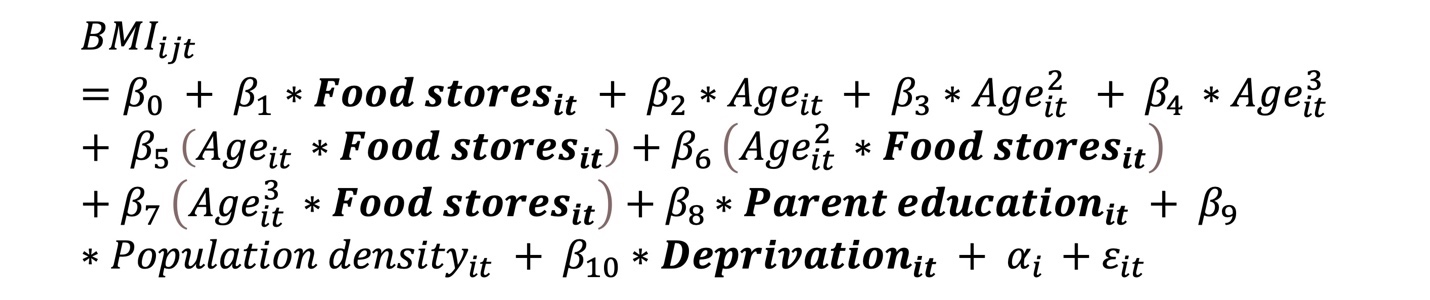
**

**Figure S2. Specification of fixed-effects linear regression model with interaction terms.**

|  | **Overall (n= 17545)** | | **1999 (n= 5568)** | | **2004 (n= 7129)** | | **2009 (n= 4848)** | |
| --- | --- | --- | --- | --- | --- | --- | --- | --- |
|  | **n** | **%** | **n** | **%** | **n** | **%** | **n** | **%** |
| **Density of small food retail stores** |  |  |  |  |  |  |  |  |
| **Quartile 1 (0.0004 - 0.14)** | 4,392 | 25.0 | 1,565 | 28.1 | 1,679 | 23.6 | 1,148 | 23.7 |
| **Quartile 2 (0.15 - 0.556)** | 4,604 | 26.2 | 1,555 | 27.9 | 1,816 | 25.5 | 1,233 | 25.4 |
| **Quartile 3 (0.561 - 2.51)** | 4,170 | 23.8 | 1,158 | 20.8 | 1,870 | 26.2 | 1,142 | 23.6 |
| **Quartile 4 (2.58 - 91.46)** | 4,379 | 25.0 | 1,290 | 23.2 | 1,764 | 24.7 | 1,325 | 27.3 |
| **Density of specialty food stores** |  |  |  |  |  |  |  |  |
| **Quartile 1 (0 - 0.013)** | 4,439 | 25.3 | 1,520 | 27.3 | 1,819 | 25.5 | 1,100 | 22.7 |
| **Quartile 2 (0.014 - 0.078)** | 4,380 | 25.0 | 1,269 | 22.8 | 1,800 | 25.2 | 1,311 | 27.0 |
| **Quartile 3 (0.08 - 0.36)** | 4,420 | 25.2 | 1,442 | 25.9 | 1,811 | 25.4 | 1,167 | 24.1 |
| **Quartile 4 (0.39 - 67.08)** | 4,306 | 24.5 | 1,337 | 24.0 | 1,699 | 23.8 | 1,270 | 26.2 |
| **Density of fruit and vegetable stores** |  |  |  |  |  |  |  |  |
| **Quartile 1 (0 - 0.0061)** | 4,407 | 25.1 | 1,386 | 24.9 | 2,012 | 28.2 | 1,009 | 20.8 |
| **Quartile 2 (0.0062 - 0.0402)** | 4,420 | 25.2 | 1,394 | 25.0 | 1,616 | 22.7 | 1,410 | 29.1 |
| **Quartile 3 (0.0404 - 0.22)** | 4,406 | 25.1 | 1,430 | 25.7 | 1,874 | 26.3 | 1,102 | 22.7 |
| **Quartile 4 (0.23 - 129.09)** | 4,312 | 24.6 | 1,358 | 24.4 | 1,627 | 22.8 | 1,327 | 27.4 |
| **Density of convenience stores** |  |  |  |  |  |  |  |  |
| **Quartile 1 (0 - 0.0001)** | 4,537 | 25.9 | 1,908 | 34.3 | 1,958 | 27.5 | 671 | 13.8 |
| **Quartile 2 (0.0002 - 0.006)** | 4,241 | 24.2 | 1,325 | 23.8 | 1,721 | 24.1 | 1,195 | 24.6 |
| **Quartile 3 (0.001 - 0.0398)** | 4,391 | 25.0 | 1,332 | 23.9 | 1,665 | 23.4 | 1,394 | 28.8 |
| **Quartile 4 (0.040 - 8.02)** | 4,376 | 24.9 | 1,003 | 18.0 | 1,785 | 25.0 | 1,588 | 32.8 |
| **Density of supermarkets** |  |  |  |  |  |  |  |  |
| **Quartile 1 (0)** | 7,388 | 42.1 | 2,836 | 50.9 | 2,824 | 39.6 | 1,728 | 35.6 |
| **Quartile 2 (0.0001 - 0.0009)** | 1,410 | 8.0 | 379 | 6.8 | 652 | 9.1 | 379 | 7.8 |
| **Quartile 3 (0.001 - 0.009)** | 4,454 | 25.4 | 1,450 | 26.0 | 1,788 | 25.1 | 1,216 | 25.1 |
| **Quartile 4 (0.01 - 1.23)** | 4,293 | 24.5 | 903 | 16.2 | 1,865 | 26.2 | 1,525 | 31.5 |
| n = observations of children | | | | | | | | |

**Table S3. Distribution of quartiles of food stores. Economic Census 1999, 2004 and 2009.**

| **Non-urban areas^a^** | | | | | | | | |  | **Urban areas^a^** | | | | | | | | | |
| --- | --- | --- | --- | --- | --- | --- | --- | --- | --- | --- | --- | --- | --- | --- | --- | --- | --- | --- | --- |
|  | **Overall (n= 8265)** | | **1999 (n= 2601)** | | **2004 (n= 3349)** | | **2009 (n= 2315)** | |  |  | **Overall (n= 9280)** | | **1999 (n= 2967)** | | **2004 (n= 3780)** | | **2009 (n= 2533)** | | |
|  | **n** | **%** | **n** | **%** | **n** | **%** | **n** | **%** |  |  | **n** | **%** | **n** | **%** | **n** | **%** | **n** | **%** |  |
| **Density of small food retail stores** |  |  |  |  |  |  |  |  |  | **Density of small food retail stores** |  |  |  |  |  |  |  |  |  |
| **Quartile 1 (0.0004 - 0.040)** | 2087 | 25.3 | 680 | 26.1 | 881 | 26.3 | 526 | 22.7 |  | **Quartile 1 (0.06 - 0.6087)** | 2508 | 27.0 | 986 | 33.2 | 1012 | 26.8 | 510 | 20.1 |  |
| **Quartile 2 (0.041 - 0.17)** | 2059 | 24.9 | 663 | 25.5 | 828 | 24.7 | 568 | 24.5 |  | **Quartile 2 (0.6095 - 2.07)** | 2133 | 23.0 | 574 | 19.4 | 859 | 22.7 | 700 | 27.6 |  |
| **Quartile 3 (0.19 - 0.36)** | 2053 | 24.8 | 673 | 25.9 | 796 | 23.8 | 584 | 25.2 |  | **Quartile 3 (2.25 - 6.09)** | 2343 | 25.3 | 684 | 23.1 | 986 | 26.1 | 673 | 26.6 |  |
| **Quartile 4 (0.39 - 3.70)** | 2066 | 25.0 | 585 | 22.5 | 844 | 25.2 | 637 | 27.5 |  | **Quartile 4 (6.15 - 91.46)** | 2296 | 24.7 | 723 | 24.4 | 923 | 24.4 | 650 | 25.7 |  |
| **Density of specialty food stores** |  |  |  |  |  |  |  |  |  | **Density of specialty food stores** |  |  |  |  |  |  |  |  |  |
| **Quartile 1 (0 - 0.0040)** | 2089 | 25.3 | 612 | 23.5 | 896 | 26.8 | 581 | 25.1 |  | **Quartile 1 (0.01 - 0.0731)** | 2332 | 25.1 | 763 | 25.7 | 944 | 25.0 | 625 | 24.7 |  |
| **Quartile 2 (0.0041 - 0.0215)** | 2069 | 25.0 | 681 | 26.2 | 847 | 25.3 | 541 | 23.4 |  | **Quartile 2 (0.0734 - 0.327)** | 2334 | 25.2 | 791 | 26.7 | 1039 | 27.5 | 504 | 19.9 |  |
| **Quartile 3 (0.0220 - 0.071)** | 2070 | 25.1 | 697 | 26.8 | 810 | 24.2 | 563 | 24.3 |  | **Quartile 3 (0.335 – 1.38)** | 2330 | 25.1 | 715 | 24.1 | 783 | 20.7 | 832 | 32.9 |  |
| **Quartile 4 (0.074 - 1.11)** | 2037 | 24.7 | 611 | 23.5 | 796 | 23.8 | 630 | 27.2 |  | **Quartile 4 (1.42 - 67.08)** | 2284 | 24.6 | 698 | 23.5 | 1,014 | 26.8 | 572 | 22.6 |  |
| **Density of fruit and vegetable stores** |  |  |  |  |  |  |  |  |  | **Density of fruit and vegetable stores** |  |  |  |  |  |  |  |  |  |
| **Quartile 1 (0 - 0.0010)** | 2072 | 25.1 | 601 | 23.1 | 1066 | 31.8 | 405 | 17.5 |  | **Quartile 1 (0 - 0.028)** | 2437 | 26.3 | 795 | 26.8 | 1003 | 26.5 | 639 | 25.2 |  |
| **Quartile 2 (0.0010 - 0.0078)** | 2183 | 26.4 | 721 | 27.7 | 688 | 20.5 | 774 | 33.4 |  | **Quartile 2 (0.031 - 0.200)** | 2254 | 24.3 | 706 | 23.8 | 1003 | 26.5 | 545 | 21.5 |  |
| **Quartile 3 (0.008 - 0.04)** | 1984 | 24.0 | 539 | 20.7 | 866 | 25.9 | 579 | 25.0 |  | **Quartile 3 (0.204 - 0.77)** | 2275 | 24.5 | 798 | 26.9 | 903 | 23.9 | 574 | 22.7 |  |
| **Quartile 4 (0.05 - 0.55)** | 2026 | 24.5 | 740 | 28.5 | 729 | 21.8 | 557 | 24.1 |  | **Quartile 4 (0.81 - 129.09)** | 2314 | 24.9 | 668 | 22.5 | 871 | 23.0 | 775 | 30.6 |  |
| **Density of convenience stores** |  |  |  |  |  |  |  |  |  | **Density of convenience stores** |  |  |  |  |  |  |  |  |  |
| **Quartile 1 (0)** | 3006 | 36.4 | 1111 | 42.7 | 1326 | 39.6 | 569 | 24.6 |  | **Quartile 1 (0 - 0.0055)** | 2373 | 25.6 | 1011 | 34.1 | 1037 | 27.4 | 325 | 12.8 |  |
| **Quartile 2 (0.0001 - 0.0013)** | 1206 | 14.6 | 383 | 14.7 | 461 | 13.8 | 362 | 15.6 |  | **Quartile 2 (0.006 - 0.0333)** | 2271 | 24.5 | 787 | 26.5 | 820 | 21.7 | 664 | 26.2 |  |
| **Quartile 3 (0.0013 - 0.0057)** | 2065 | 25.0 | 645 | 24.8 | 855 | 25.5 | 565 | 24.4 |  | **Quartile 3 (0.0340 - 0.1031)** | 2346 | 25.3 | 712 | 24.0 | 1063 | 28.1 | 571 | 22.5 |  |
| **Quartile 4 (0.006 - 0.14)** | 1988 | 24.1 | 462 | 17.8 | 707 | 21.1 | 819 | 35.4 |  | **Quartile 4 (0.1033 - 8.02)** | 2290 | 24.7 | 457 | 15.4 | 860 | 22.8 | 973 | 38.4 |  |
| **Density of supermarkets^b^** |  |  |  |  |  |  |  |  |  | **Density of supermarkets** |  |  |  |  |  |  |  |  |  |
| **Quartile 1 (0)** | 5662 | 68.5 | 1908 | 73.4 | 2287 | 68.3 | 1467 | 63.4 |  | **Quartile 1 (0 - 0.0011)** | 2362 | 25.5 | 1144 | 38.6 | 860 | 22.8 | 358 | 14.1 |  |
| **Quartile 2** |  |  |  |  |  |  |  |  |  | **Quartile 2 (0.0011 - 0.078)** | 2459 | 26.5 | 736 | 24.8 | 1055 | 27.9 | 668 | 26.4 |  |
| **Quartile 3 (0.0001 - 0.0005)** | 580 | 7.0 | 187 | 7.2 | 245 | 7.3 | 148 | 6.4 |  | **Quartile 3 (0.0083 - 0.023)** | 2154 | 23.2 | 426 | 14.4 | 1072 | 28.4 | 656 | 25.9 |  |
| **Quartile 4 (0.0006 - 0.02)** | 2023 | 24.5 | 506 | 19.5 | 817 | 24.4 | 700 | 30.2 |  | **Quartile 4 (0.024 - 1.23)** | 2305 | 24.8 | 661 | 22.3 | 793 | 21.0 | 851 | 33.6 |  |
| n = observations of children | | | | | | | | | | | | | | | | | | | |
| ^a^ Urbanization is defined by the population in 2010: urban areas are municipalities that belong to a city with more than 100,000 residents as defined by SALURBAL [3,4], while non-urban areas refer to all other municipalities (see Methods section). | | | | | | | | | | | | | | | | | | | |
| ^b^ Due to distribution of density of supermarkets, the second quartile was not able to be estimated. | | | | | | | | | | | | | | | | | | | |

**Table S4. Distribution of quartiles of food stores by urbanization. Economic Census 1999, 2004 and 2009.**

|  | **Overall** | | |  | **Non-urban areas^a^** | | |  | **Urban areas^a^** | | |
| --- | --- | --- | --- | --- | --- | --- | --- | --- | --- | --- | --- |
|  | **β^b^** | **95%CI** | **P-value** |  | **β^b^** | **95%CI** | **P-value** |  | **β^b^** | **95%CI** | **P-value** |
| **Food store type^c^** |  |  |  |  |  |  |  |  |  |  |  |
| **Proportion of small food retail stores** | 0.036 | -0.026 , 0.098 | 0.255 |  | 0.039 | -0.026 , 0.103 | 0.239 |  | 0.117 | -0.138 , 0.372 | 0.368 |
| **Proportion of specialty food stores** | -0.063 | -0.144 , 0.017 | 0.124 |  | -0.058 | -0.140 , 0.024 | 0.165 |  | -0.266 | -0.745 , 0.214 | 0.277 |
| **Proportion of fruit and vegetable stores** | 0.009 | -0.105 , 0.123 | 0.881 |  | 0.024 | -0.096 , 0.145 | 0.691 |  | -0.156 | -0.509 , 0.198 | 0.389 |
| **Proportion of convenience stores** | -0.019 | -0.138 , 0.101 | 0.760 |  | -0.052 | -0.180 , 0.076 | 0.424 |  | 0.077 | -0.341 , 0.494 | 0.719 |
| **Proportion of supermarkets** | 1.322 | 0.139 , 2.506 | **0.029** |  | 1.292 | 0.054 , 2.530 | **0.041** |  | 2.430 | -1.568 , 6.428 | 0.234 |
| β = Coefficients of the regressors; 95% CI = 95% Confidence Interval. | | | | | | | | | | | |
| Overall: Number of observations = 17545; Number of participants = 7507. Non-urban areas: Number of observations = 8265; number of participants = 3544. Urban areas: Number of observations = 9280; number of participants = 4049. | | | | | | | | | | | |
| ^a^ Urbanization is defined by the population in 2010: urban areas are municipalities that belong to a city with more than 100,000 residents as defined by SALURBAL [3,4], while non-urban areas refer to all other municipalities (see Methods section). | | | | | | | | | | | |
| ^b^ Five linear fixed-effects regression models (one per food store type) adjusted for age, age2, age3, parental education level, population density, and socioeconomic deprivation. | | | | | | | | | | | |
| ^c^ Exposure variable = Proportion of food stores at the municipality level = Number of each food store category over the total of food stores, was rescaled it by dividing it by 10, to represent 10% increment. | | | | | | | | | | | |

**Table S5. Within-person mean difference in body mass index associated with within-person mean differences in proportion of food stores.**

|  | **Density of small food retail stores** | | | | | | |
| --- | --- | --- | --- | --- | --- | --- | --- |
|  | **Quartile 1** | **Quartile 2** | | **Quartile 3** | | **Quartile 4** | |
| **Age** | **β** | **β** | **95%CI** | **β** | **95%CI** | **β** | **95%CI** |
| 5 | Ref. | -0.08 | -0.61 , 0.45 | 0.15 | -0.44 , 0.74 | -0.03 | -0.73 , 0.67 |
| 6 | Ref. | 0.04 | -0.36 , 0.44 | 0.17 | -0.30 , 0.64 | 0.07 | -0.53 , 0.68 |
| 7 | Ref. | 0.10 | -0.25 , 0.45 | 0.17 | -0.26 , 0.60 | 0.17 | -0.40 , 0.75 |
| 8 | Ref. | 0.12 | -0.22 , 0.45 | 0.16 | -0.26 , 0.58 | 0.26 | -0.31 , 0.83 |
| 9 | Ref. | 0.09 | -0.24 , 0.42 | 0.14 | -0.28 , 0.55 | 0.33 | -0.23 , 0.90 |
| 10 | Ref. | 0.04 | -0.29 , 0.36 | 0.10 | -0.31 , 0.51 | 0.39 | -0.17 , 0.94 |
| 11 | Ref. | -0.04 | -0.36 , 0.27 | 0.05 | -0.35 , 0.45 | 0.41 | -0.14 , 0.96 |
| 12 | Ref. | -0.13 | -0.44 , 0.18 | 0.00 | -0.40 , 0.39 | 0.40 | -0.15 , 0.95 |
| 13 | Ref. | -0.22 | -0.54 , 0.09 | -0.07 | -0.47 , 0.33 | 0.36 | -0.19 , 0.91 |
| 14 | Ref. | -0.31 | -0.64 , 0.01 | -0.14 | -0.55 , 0.27 | 0.27 | -0.28 , 0.83 |
| 15 | Ref. | -0.39 | -0.72 , -0.06 | -0.21 | -0.63 , 0.21 | 0.14 | -0.42 , 0.71 |
| 16 | Ref. | -0.44 | -0.78 , -0.11 | -0.29 | -0.71 , 0.13 | -0.04 | -0.61 , 0.53 |
| 17 | Ref. | -0.47 | -0.82 , -0.11 | -0.37 | -0.80 , 0.07 | -0.28 | -0.85 , 0.30 |
| 18 | Ref. | -0.45 | -0.87 , -0.03 | -0.45 | -0.94 , 0.04 | -0.58 | -1.19 , 0.04 |
| 19 | Ref. | -0.39 | -0.97 , 0.18 | -0.53 | -1.15 , 0.10 | -0.94 | -1.67 , -0.21 |
| β = Coefficients of the regressors; 95% CI = 95% Confidence Interval | | | | | | | |

**Table S6a. Marginal effects considering interaction between age and quartiles of density of small food retail stores on body mass index trajectories.**

|  | **Density of specialty food stores** | | | | | | |
| --- | --- | --- | --- | --- | --- | --- | --- |
|  | **Quartile 1** | **Quartile 2** | | **Quartile 3** | | **Quartile 4** | |
| **Age** | **β** | **β** | **95%CI** | **β** | **95%CI** | **β** | **95%CI** |
| 5 | Ref. | -0.16 | -0.70 , 0.38 | 0.21 | -0.36 , 0.79 | -0.24 | -0.99 , 0.52 |
| 6 | Ref. | -0.13 | -0.53 , 0.26 | 0.07 | -0.40 , 0.53 | -0.21 | -0.88 , 0.46 |
| 7 | Ref. | -0.12 | -0.46 , 0.22 | -0.05 | -0.47 , 0.38 | -0.16 | -0.80 , 0.48 |
| 8 | Ref. | -0.12 | -0.45 , 0.21 | -0.13 | -0.55 , 0.30 | -0.10 | -0.74 , 0.53 |
| 9 | Ref. | -0.12 | -0.44 , 0.20 | -0.19 | -0.61 , 0.23 | -0.04 | -0.67 , 0.59 |
| 10 | Ref. | -0.13 | -0.44 , 0.18 | -0.23 | -0.65 , 0.19 | 0.02 | -0.61 , 0.64 |
| 11 | Ref. | -0.15 | -0.45 , 0.16 | -0.26 | -0.68 , 0.16 | 0.06 | -0.56 , 0.68 |
| 12 | Ref. | -0.16 | -0.46 , 0.13 | -0.28 | -0.70 , 0.14 | 0.09 | -0.52 , 0.71 |
| 13 | Ref. | -0.18 | -0.48 , 0.12 | -0.30 | -0.73 , 0.13 | 0.10 | -0.52 , 0.72 |
| 14 | Ref. | -0.20 | -0.51 , 0.12 | -0.32 | -0.76 , 0.11 | 0.07 | -0.56 , 0.69 |
| 15 | Ref. | -0.21 | -0.53 , 0.11 | -0.35 | -0.80 , 0.09 | 0.00 | -0.63 , 0.62 |
| 16 | Ref. | -0.22 | -0.55 , 0.11 | -0.40 | -0.85 , 0.06 | -0.12 | -0.75 , 0.51 |
| 17 | Ref. | -0.23 | -0.57 , 0.12 | -0.46 | -0.93 , 0.01 | -0.30 | -0.93 , 0.34 |
| 18 | Ref. | -0.22 | -0.63 , 0.19 | -0.55 | -1.08 , -0.03 | -0.54 | -1.21 , 0.14 |
| 19 | Ref. | -0.21 | -0.77 , 0.35 | -0.67 | -1.33 , -0.01 | -0.84 | -1.62 , -0.07 |
| β = Coefficients of the regressors; 95% CI = 95% Confidence Interval | | | | | | | |

**Table S6b. Marginal effects considering interaction between age and quartiles of density of specialty food stores on body mass index trajectories.**

|  | **Density of fruit and vegetable stores** | | | | | | |
| --- | --- | --- | --- | --- | --- | --- | --- |
|  | **Quartile 1** | **Quartile 2** | | **Quartile 3** | | **Quartile 4** | |
| **Age** | **β** | **β** | **95%CI** | **β** | **95%CI** | **β** | **95%CI** |
| 5 | Ref. | -0.03 | -0.55 , 0.49 | -0.56 | -1.11 , -0.01 | -0.85 | -1.48 , -0.21 |
| 6 | Ref. | -0.22 | -0.59 , 0.15 | -0.60 | -1.02 , -0.17 | -0.87 | -1.40 , -0.34 |
| 7 | Ref. | -0.35 | -0.67 , -0.04 | -0.62 | -1.01 , -0.24 | -0.85 | -1.34 , -0.36 |
| 8 | Ref. | -0.44 | -0.74 , -0.14 | -0.65 | -1.02 , -0.28 | -0.81 | -1.29 , -0.33 |
| 9 | Ref. | -0.49 | -0.78 , -0.19 | -0.67 | -1.04 , -0.30 | -0.76 | -1.24 , -0.28 |
| 10 | Ref. | -0.51 | -0.79 , -0.22 | -0.69 | -1.06 , -0.33 | -0.70 | -1.17 , -0.23 |
| 11 | Ref. | -0.51 | -0.78 , -0.24 | -0.72 | -1.07 , -0.36 | -0.65 | -1.12 , -0.18 |
| 12 | Ref. | -0.50 | -0.77 , -0.23 | -0.74 | -1.09 , -0.38 | -0.62 | -1.08 , -0.15 |
| 13 | Ref. | -0.48 | -0.75 , -0.21 | -0.76 | -1.12 , -0.39 | -0.62 | -1.09 , -0.15 |
| 14 | Ref. | -0.47 | -0.76 , -0.18 | -0.78 | -1.16 , -0.41 | -0.66 | -1.13 , -0.18 |
| 15 | Ref. | -0.47 | -0.77 , -0.18 | -0.81 | -1.19 , -0.43 | -0.75 | -1.23 , -0.27 |
| 16 | Ref. | -0.50 | -0.80 , -0.19 | -0.84 | -1.23 , -0.45 | -0.90 | -1.39 , -0.42 |
| 17 | Ref. | -0.55 | -0.88 , -0.23 | -0.88 | -1.29 , -0.47 | -1.13 | -1.63 , -0.63 |
| 18 | Ref. | -0.65 | -1.04 , -0.26 | -0.92 | -1.39 , -0.45 | -1.44 | -1.99 , -0.90 |
| 19 | Ref. | -0.80 | -1.35 , -0.24 | -0.97 | -1.58 , -0.36 | -1.85 | -2.52 , -1.18 |
| β = Coefficients of the regressors; 95% CI = 95% Confidence Interval | | | | | | | |

**Table S6c. Marginal effects considering interaction between age and quartiles of density of fruit and vegetable stores on body mass index trajectories.**

|  | **Density of convenience stores** | | | | | | |
| --- | --- | --- | --- | --- | --- | --- | --- |
|  | **Quartile 1** | **Quartile 2** | | **Quartile 3** | | **Quartile 4** | |
| **Age** | **β** | **β** | **95%CI** | **β** | **95%CI** | **β** | **95%CI** |
| 5 | Ref. | -0.29 | -0.78 , 0.20 | -0.14 | -0.61 , 0.33 | -0.47 | -0.98 , 0.05 |
| 6 | Ref. | -0.18 | -0.52 , 0.16 | 0.04 | -0.28 , 0.35 | -0.19 | -0.57 , 0.18 |
| 7 | Ref. | -0.08 | -0.36 , 0.20 | 0.16 | -0.09 , 0.42 | 0.03 | -0.29 , 0.35 |
| 8 | Ref. | 0.01 | -0.26 , 0.27 | 0.24 | -0.01 , 0.48 | 0.21 | -0.10 , 0.51 |
| 9 | Ref. | 0.08 | -0.18 , 0.34 | 0.27 | 0.03 , 0.51 | 0.33 | 0.03 , 0.64 |
| 10 | Ref. | 0.13 | -0.12 , 0.38 | 0.26 | 0.03 , 0.49 | 0.41 | 0.12 , 0.70 |
| 11 | Ref. | 0.16 | -0.07 , 0.40 | 0.22 | 0.01 , 0.44 | 0.45 | 0.17 , 0.73 |
| 12 | Ref. | 0.18 | -0.06 , 0.42 | 0.16 | -0.05 , 0.38 | 0.43 | 0.16 , 0.71 |
| 13 | Ref. | 0.17 | -0.08 , 0.42 | 0.09 | -0.14 , 0.31 | 0.37 | 0.10 , 0.65 |
| 14 | Ref. | 0.13 | -0.13 , 0.40 | 0.00 | -0.24 , 0.24 | 0.27 | -0.02 , 0.56 |
| 15 | Ref. | 0.07 | -0.21 , 0.35 | -0.09 | -0.35 , 0.16 | 0.11 | -0.19 , 0.41 |
| 16 | Ref. | -0.03 | -0.32 , 0.26 | -0.18 | -0.45 , 0.08 | -0.09 | -0.40 , 0.22 |
| 17 | Ref. | -0.15 | -0.47 , 0.16 | -0.26 | -0.56 , 0.03 | -0.33 | -0.66 , 0.00 |
| 18 | Ref. | -0.31 | -0.71 , 0.08 | -0.32 | -0.71 , 0.06 | -0.63 | -1.03 , -0.22 |
| 19 | Ref. | -0.51 | -1.09 , 0.07 | -0.36 | -0.93 , 0.21 | -0.97 | -1.53 , -0.40 |
| β = Coefficients of the regressors; 95% CI = 95% Confidence Interval | | | | | | | |

**Table S6d. Marginal effects considering interaction between age and quartiles of density of convenience stores on body mass index trajectories.**

|  | **Density of supermarkets** | | | | | | |
| --- | --- | --- | --- | --- | --- | --- | --- |
|  | **Quartile 1** | **Quartile 2** | | **Quartile 3** | | **Quartile 4** | |
| **Age** | **β** | **β** | **95%CI** | **β** | **95%CI** | **β** | **95%CI** |
| 5 | Ref. | -0.22 | -0.94 , 0.50 | 0.45 | 0.02 , 0.87 | -0.09 | -0.58 , 0.40 |
| 6 | Ref. | -0.17 | -0.68 , 0.34 | 0.44 | 0.16 , 0.73 | -0.03 | -0.39 , 0.34 |
| 7 | Ref. | -0.12 | -0.54 , 0.30 | 0.44 | 0.21 , 0.67 | 0.07 | -0.25 , 0.38 |
| 8 | Ref. | -0.07 | -0.47 , 0.33 | 0.44 | 0.22 , 0.66 | 0.17 | -0.13 , 0.47 |
| 9 | Ref. | -0.03 | -0.42 , 0.36 | 0.44 | 0.22 , 0.66 | 0.28 | -0.01 , 0.57 |
| 10 | Ref. | 0.00 | -0.37 , 0.38 | 0.43 | 0.22 , 0.64 | 0.38 | 0.10 , 0.66 |
| 11 | Ref. | 0.02 | -0.33 , 0.37 | 0.41 | 0.21 , 0.61 | 0.45 | 0.19 , 0.72 |
| 12 | Ref. | 0.02 | -0.32 , 0.37 | 0.38 | 0.19 , 0.57 | 0.50 | 0.24 , 0.76 |
| 13 | Ref. | 0.00 | -0.34 , 0.35 | 0.34 | 0.14 , 0.54 | 0.50 | 0.24 , 0.76 |
| 14 | Ref. | -0.04 | -0.40 , 0.32 | 0.28 | 0.06 , 0.49 | 0.45 | 0.18 , 0.71 |
| 15 | Ref. | -0.11 | -0.48 , 0.27 | 0.20 | -0.02 , 0.43 | 0.33 | 0.05 , 0.60 |
| 16 | Ref. | -0.21 | -0.59 , 0.18 | 0.10 | -0.13 , 0.33 | 0.13 | -0.15 , 0.40 |
| 17 | Ref. | -0.34 | -0.74 , 0.07 | -0.02 | -0.27 , 0.23 | -0.16 | -0.44 , 0.13 |
| 18 | Ref. | -0.51 | -1.00 , -0.02 | -0.17 | -0.50 , 0.16 | -0.54 | -0.89 , -0.20 |
| 19 | Ref. | -0.72 | -1.42 , -0.01 | -0.35 | -0.84 , 0.14 | -1.04 | -1.53 , -0.56 |
| β = Coefficients of the regressors; 95% CI = 95% Confidence Interval | | | | | | | |

**Table S6e. Marginal effects considering interaction between age and quartiles of density of supermarkets on body mass index trajectories.**

**References for supplementary material**

1. Instituto Nacional de Estadística Geografía e Informática (INEGI). Directorio Estadístico Nacional de Unidades Económicas. DENUE Interactivo 11/2017. Documento metodológico [Internet]. 2017 [cited 2021 Aug 19]. Available from: https://www.inegi.org.mx/app/biblioteca/ficha.html?upc=702825097240

2. StataCorp. Stata 14 Base Reference Manual. College Station, TX; 2015.

3. Diez Roux A V., Slesinski SC, Alazraqui M, Caiaffa WT, Frenz P, Jordán Fuchs R, et al. A Novel International Partnership for Actionable Evidence on Urban Health in Latin America: LAC‐Urban Health and SALURBAL. Glob Challenges. Wiley; 2019;3:1800013.

4. Quistberg DA, Diez Roux A V., Bilal U, Moore K, Ortigoza A, Rodriguez DA, et al. Building a Data Platform for Cross-Country Urban Health Studies: the SALURBAL Study. J Urban Heal. Springer Science and Business Media Deutschland GmbH; 2019;96:311–37.
